# Thermophysical properties of 5-methylfurfural with alcohol additives: Toward efficient and sustainable biofuel blends

**DOI:** 10.1016/j.isci.2025.114591

**Published:** 2025-12-31

**Authors:** Mohammad Almasi, Morteza Vatanparast

**Affiliations:** 1 Department of Applied Chemistry, Faculty of Science, Malayer University, Malayer 65174, Iran

**Keywords:** Electrochemical energy production, Computational molecular modelling, Energy materials

## Abstract

The transition toward sustainable energy systems requires renewable fuel alternatives with optimized combustion performance and reduced emissions. 5-Methylfurfural (MFF), a lignocellulosic biomass-derived furanic compound, has emerged as a promising biofuel precursor and blending. In this study, we investigated the thermophysical properties and molecular interactions of binary mixtures of MFF with short-to medium-chain 2-alkanols (C_3_–C_6_), combining experimental measurements of density and viscosity with molecular dynamics (MD) simulations. Our findings reveal strong hydrogen-bond interactions between MFF’s aldehyde group and the hydroxyl groups of short-chain alcohols, leading to compact molecular packing, enhanced volatility, and improved combustion efficiency. Conversely, longer-chain alcohols introduce steric hindrance that disrupts hydrogen-bonding networks, increasing free volume and diffusivity, thereby influencing ignition delay and miscibility with conventional fuels. These results provide molecular-level insights into customizing viscosity, density, and volatility of renewable fuel blends, thereby enabling the design of advanced biofuels with improved engine compatibility and environmental performance.

## Highlights

• Hydrogen bonding governs MFF/alcohol blend thermophysical behavior
• Short-chain alcohols enhance volatility and packing for SI engines
• Long-chain alcohols improve miscibility and stability for CI engines
• Experimental and MD results show strong quantitative agreement

## Graphical abstract

**Figure:**
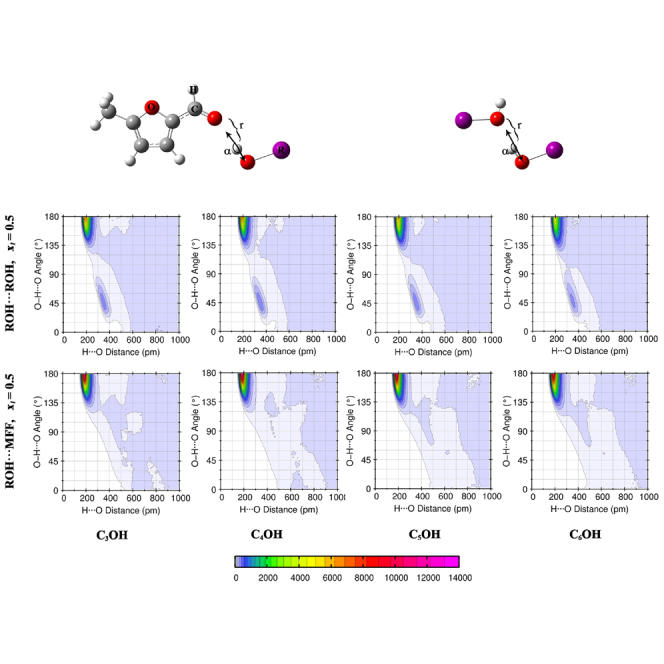

## Introduction

The global shift toward low-carbon energy systems has intensified interest in biomass-derived fuels that can reduce reliance on fossil resources and mitigate greenhouse gas emissions. Among the most promising bio-based intermediates is 5-methylfurfural (MFF), a C_6_ furanic compound obtained from lignocellulosic biomass, which serves as a versatile platform for both chemical synthesis and renewable fuel development.^1^^,^^2^ Its unique structure, combining a furan ring and an aldehyde group, allows upgrading into high-performance biofuels such as 2,5-dimethylfuran (DMF) and 2,5-dimethyltetrahydrofuran (DMTHF), compounds known for their excellent energy densities and combustion properties.^3^^,^^4^ Additionally, catalytic C–C coupling of MFF enables the production of longer-chain hydrocarbons, providing routes to jet and diesel fuel alternatives.^5^^,^^6^ Beyond its role as a precursor, MFF itself is a candidate for direct fuel blending due to its oxygenated nature, which supports cleaner combustion and reduced soot emissions. Its compatibility with alcohol additives further enhances its applicability, as alcohols can improve volatility, miscibility, and atomization in combustion environments.^7^^,^^8^^,^^9^ Short-chain alcohols such as 2-propanol are particularly attractive due to their high oxygen content, volatility, and ability to form strong hydrogen bonds with MFF’s carbonyl group, thereby improving ignition and reducing particulate matter formation in spark ignition (SI) engines.^8^^,^^9^ Conversely, long-chain alcohols such as 2-hexanol, with higher energy density and cetane numbers, exhibit better miscibility with conventional fuels and enhance combustion stability in compression ignition (CI) engines.^10^^,^^11^^,^^12^^,^^13^^,^^14^

Several recent studies show that combining excess volume and viscosity data can clearly separate packing versus specific interaction effects. For example, Verma et al. measured viscosity deviations for butanol + aromatic hydrocarbon mixtures and, by coupling these with excess molar volumes, concluded that aromatic rings “depolymerize” the hydrogen-bonded butanol network – the strength of the resulting electron donor-acceptor interactions depends on the aromatic’s π-electron density.^15^ Similarly, Verma et al. found that diethyl ether + cyclohexane gave positive excess volume and negative ultrasonic speed deviation (no specific interactions), whereas diethyl ether + benzene/toluene gave negative excess volume and positive Δu (speed of sound), revealing strong O–π interactions.^16^ These contrasting signatures (expansion with lower viscosity vs. contraction with higher viscosity) directly correspond to the two contributions, simple volumetric packing versus specific associative interactions.^17^

Understanding the molecular interactions governing MFF/alcohol mixtures is therefore crucial for adapting blend properties to meet specific engine performance and environmental targets. Experimental characterization of density and viscosity, coupled with molecular dynamics (MD) simulations, provides an integrated framework to elucidate hydrogen bonding, molecular packing, and free-volume effects in these systems. By bridging molecular-scale insights with macroscopic thermophysical behavior, this study establishes a predictive approach to optimize biofuel blends that align with the goals of efficiency, reduced emissions, and compatibility with existing engine technologies.

## Results and discussion

### Density

**Figure 1:**
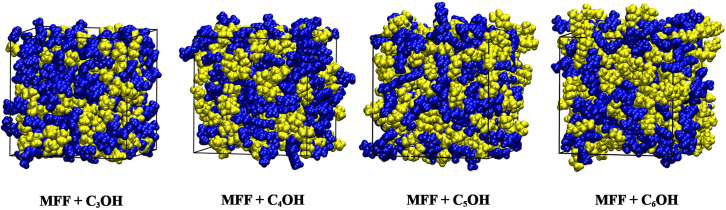
Final snapshot for the system of x_1_ = 0.5 MFF-blue, ROH-yellow.

The experimental and molecular dynamics simulated densities (ρ) for 5-methyl furfural (MFF) with 2-propanol (C_3_OH), 2-butanol (C_4_OH), 2-pentanol (C_5_OH), and 2-hexanol (C_6_OH) are presented in Figure 1. The density values are plotted as a mole fraction of MFF (x_1_) at T(K) = 293.15.

**Figure 2:**
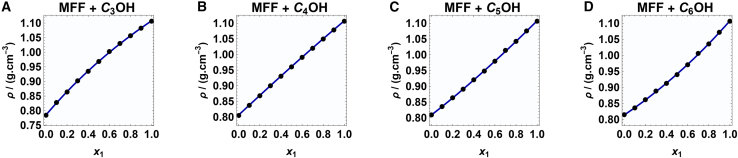
Experimental and simulated data of density of MFF and 2-alkanol (A–D) Density vs. mole fraction (x_1_) for binary systems of MFF and 2-alkanol: experimental data (solid lines) and MD simulations (points).

In all four systems (Figures 2A–2D), density shows a nearly linear dependence on composition, increasing gradually with increasing MFF content. This trend reflects the inherently higher density of MFF compared to the corresponding alcohols, which leads to an increase in mixture density as MFF becomes the dominant component. The observed agreement between simulation and experimental results demonstrates the strong predictive capability of the employed force field and validates the computational methodology used in this study.

### Hydrogen bond

**Figure 3:**
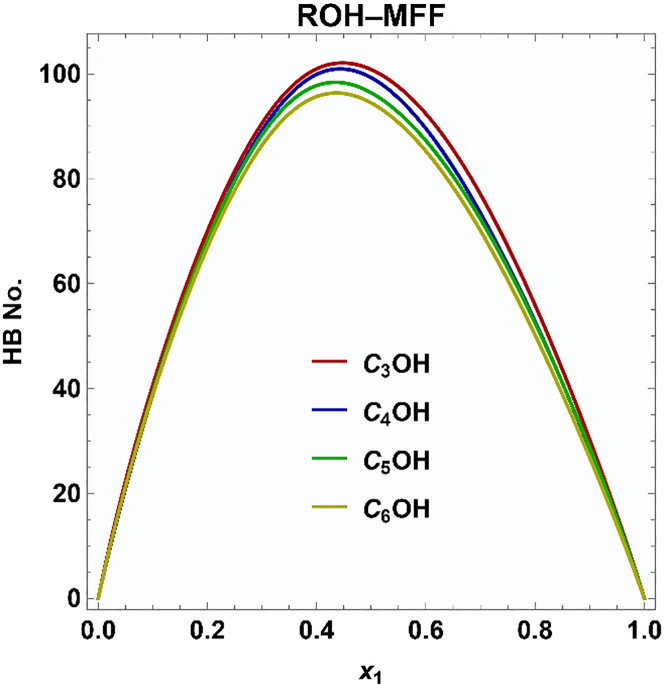
Number of hydrogen bonds between 5-methyl furfural and alcohols

Figure 3 depicts the variation in the number of hydrogen bonds between MFF and alcohol as a function of the MFF mole fraction in the MFF + 2-alkanol binary mixtures, as obtained from molecular dynamics simulations. ROH···MFF hydrogen bonds increase with MFF mole fraction, reaching a maximum near equimolar compositions and then declining as the alcohol concentration becomes insufficient to sustain significant hydrogen bonding. A comparative analysis across the four systems reveals that the extent of ROH···MFF interactions displays a decreasing trend with the elongation of the alcohol’s alkyl chain. This can be ascribed to the steric hindrance of longer alkyl chains, which can hinder optimal hydrogen bond formation with the MFF.

### Radial distribution function analysis

**Figure 4:**
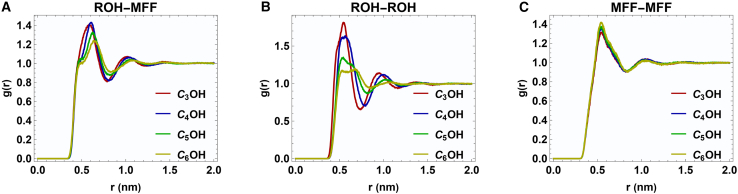
Center-of-mass RDF profiles for the binary mixtures (A–C) Center-of-mass RDF profiles at x_1_ = 0.5 for ROH-MFF, ROH-ROH, and MFF-MFF interactions.

The center-of-mass radial distribution functions were computed at an equimolar composition (x_1_ = 0.5) to elucidate the molecular interactions in the MFF + ROH systems. Figure 4 presents the resulting g(r) profiles for (i) alcohol-methyl 5-methyl furfural (ROH-MFF), (ii) alcohol-alcohol (ROH-ROH), and (iii) 5-methyl furfural-5-methyl furfural (MFF-MFF) pair correlations.

In Figure 4A, all four mixtures display a sharp first peak in the ROH-MFF radial distribution function between 0.50 and 0.65 nm. When the alkyl chain length of the alcohol is extended from C_3_ to C_6_, a clear trends emerge: the peak maximum shifts toward longer distances (r). These observations indicate that the shorter-chain alcohols (particularly C_3_OH and C_4_OH) form tighter local packing. Conversely, the larger 2-pentanol (C_5_OH) and 2-hexanol (C_6_OH) molecules have greater steric hindrance, resulting in less frequent and weaker hydrogen bonding with MFF, as reflected by reduced peak intensities and a shift of the maxima to longer distances.

In Figure 4B, the primary peak at r ≈ 0.55 nm arises from hydrogen bonds between neighboring alcohol molecules. The peak intensity follows the order: g(r) C_3_OH > g(r) C_4_OH > g(r) C_5_OH > g(r) C_6_OH, consistent with the dilution of the alcohol network by MFF and increased chain-chain steric effects in bulkier alcohols. Additionally, secondary peaks emerging at approximately 0.9–1.0 nm indicate longer-range structuring of the alcohol hydrogen-bond network.

The MFF-MFF g(r) functions show a distinct first peak at roughly r ≈ 0.54 nm, associated with π–π stacking or edge-to-face interactions between furan rings (Figure 4C). This feature is nearly invariant across all alcohol environments, indicating that MFF self-association is only weakly perturbed by the different alcohols at the equimolar composition.

**Figure 5:**
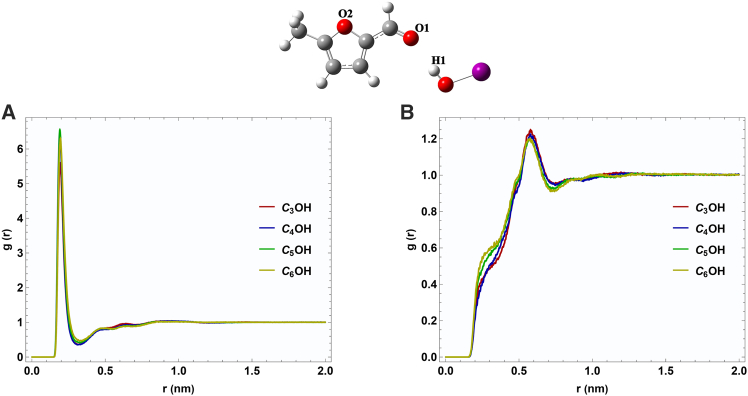
RDF for hydrogen bonding in two oxygen position of MFF RDFs *g*(r) for (A) MFF carbonyl oxygen O1–alcohol hydrogen H1 and (B) MFF furan oxygen O2–alcohol hydrogen H1, at x_1_ = 0.5.

Figure 5 demonstrates the site-site radial distribution functions (RDFs) between the hydrogen-bond donor atom H1 of each alcohol and the two potential acceptor sites of 5-methylfurfural (MFF) at x1 = 0.5). In Figure 5A, all four alcohols exhibit a sharp peak at around 0.19–0.20 nm, with maximum *g*(r) values of approximately 5.5–6.5. This intense, short-range peak is characteristic of a strong, directional hydrogen bond between the alcohol OH proton and the MFF carbonyl oxygen. The near-identical position and height of the first peak for C_3_OH, C_4_OH, C_5_OH, and C_6_OH indicate that the immediate hydrogen-bonding environment at O1 is essentially independent of the alcohol chain length. Beyond the first shell, *g*(r) rapidly decays to unity, showing no significant medium-range structuring. Figure 5B presents the RDFs for O2–H1. Here, the first peak appears at a slightly longer distance (∼0.25 nm) and is much less intense, indicating only weak, non-specific interactions of the alcohol OH with the furan oxygen. As with O1, the position of this feature is invariant with chain length, but the modest increase in peak height from 2-propanol to 2-hexanol suggests a slight enhancement of van der Waals contacts for larger alcohols in the furan ring’s vicinity.

Overall, the data demonstrate that MFF’s carbonyl oxygen is the primary hydrogen-bond acceptor in these mixtures, forming strong, localized bonds with the alcohol OH group.

### Combined distribution functions

**Figure 6:**
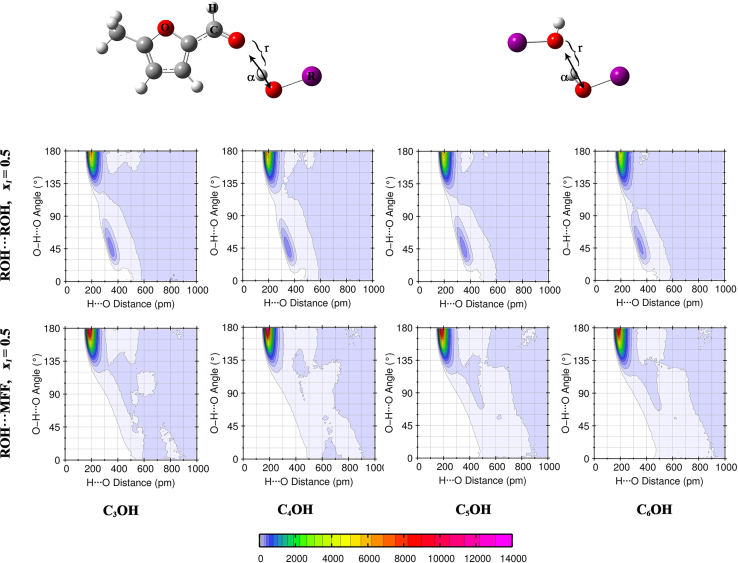
Combined distribution functions for H···O distances and O–H···O angles at x_1_ = 0.5

In order to characterize the hydrogen bonding in alcohol-alcohol self-association and alcohol-MFF cross-association in our mixtures, we extracted two-dimensional combined distribution functions (CDFs) of the H···O distance versus the O–H···O angle at x_1_ = 0.5. Figure 6 shows these maps, with the top row corresponding to H···OH contacts (alcohol self-association) and the bottom row to OH···O MFF contacts (alcohol-MFF hydrogen bonds).

All four alcohols display a sharp, intense ridge centered at H···O ≈ 200 p.m. and O–H···O angle ≈180°, a characteristic of strong linear hydrogen bonds between alcohol molecules. The color scale shows that this region is most highly populated for 2-propanol and 2-butanol and gradually diminishes for 2-pentanol and 2-hexanol. This trend reflects the well-known decrease in self-aggregation propensity as the alkyl chain lengthens and steric hindrance grows.

A distinct peak is seen at H···O ≃ 180–200 p.m. and angle ≃ 160–180°, indicating that each alcohol also forms directional hydrogen bonds to the carbonyl oxygen of MFF (bottom row of Figure 6). As the alcohol chain grows from C_3_OH to C_6_OH, the intensity of the cross-association peak further decreases, suggesting that steric bulk around the OH group hinders its ability to approach and align with the MFF carbonyl.

### Analysis of orientation

**Figure 7:**
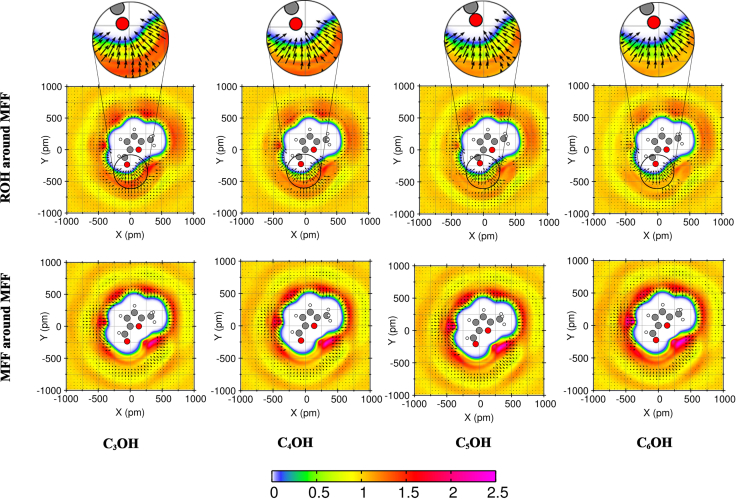
Plane projection analysis of MFF and alcohol (ROH) systems

To visualize the spatial organization and preferred orientation of the two species around each other, we performed plane projection analyses at x_1_ = 0.5 (depicted in Figure 7). The colorimetric scale employed in these projections quantitatively represents the average particle density of the molecule at the given position relative to uniform density. This analytical approach enables the characterization of preferred molecular orientations and the dominant intermolecular interactions that dictate the mesoscopic structure of these binary systems.

For alcohol distribution around MFF (top row), the highest probability density (red regions, density) is localized immediately above and below the MFF carbonyl oxygen (O1), indicating that alcohol molecules preferentially approach this site. The orientation vectors in the high-density O1 region point directly toward the carbonyl, with O–H bonds aligned nearly perpendicular to the MFF plane, again confirming strong, linear hydrogen bonds. With increasing alkyl chain length from C_3_OH to C_6_OH, the maximum density slightly decreases, indicating that the steric hindrance of longer chains reduces the tightness of alcohol around O1. In the bottom row of Figure 7, the two-dimensional distribution of MFF molecules around a reference MFF molecule is depicted. This analysis provides insight into the self-association behavior of MFF in alcoholic environments. As observed in the density maps, MFF molecules are distributed relatively homogeneously around each other, with no strong evidence of directional aggregation. However, a noticeable decrease in density appears around the carbonyl oxygen (O1) site. This reduction can be attributed to the fact that the O1 region preferentially forms hydrogen bonds with alcohol molecules, thereby reducing the likelihood of MFF-MFF interactions in that area. The reason for this nearly uniform distribution is the absence of strong hydrogen bonding between MFF molecules themselves. The polar functional groups of MFF tend to engage more strongly with the hydroxyl groups of alcohols, leaving only weak residual electrostatic or dipole-dipole interactions between MFF molecules.

### Mean-squared displacements of species

**Figure 8:**
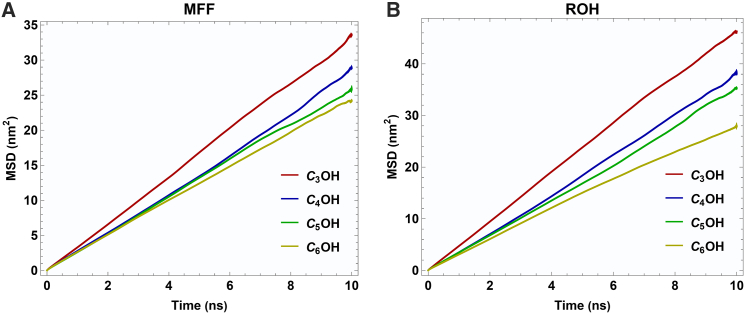
Plots of mean squared displacement (MSD) vs. time, x_1_ = 0.5

Figure 8 shows the mean-squared displacement (MSD) of 5-methylfurfural (MFF) and the four secondary alcohols in equimolar (x_1_ = 0.5) binary mixtures as a function of simulation time. To further quantitatively assess the relative mobilities of each component, diffusion coefficients (D) were calculated and are presented in Table 1.

**Table 1:** Calculated diffusion coefficients D_12_ (10^−6^ m^2^ S^−1^) of species in mole fraction x_1_ = 0.5

Alcohols	Diffusion coefficients of alcohols in x_1_ = 0.5 of	Diffusion coefficients of Me in x_1_ = 0.5 of
2-Propanol	0.00079	0.00056
2-Butanol	0.00060	0.00044
2-Pentanol	0.00056	0.00044
2-Hexanol	0.00050	0.00041

For both components, D decreases as the alcohol alkyl chain lengthens. The drop from 2-propanol to 2-butanol is most pronounced (∼24% for the alcohol), after which the reduction becomes less steep (only ∼7% decrease from 2-butanol to 2-pentanol). This reflects the increasing van der Waals interactions and steric hindrance in longer chains.

At each chain length, the diffusion coefficient of the alcohol is greater than that of MFF, which indicates that steric hindrance has a larger influence on MFF.

### Void analysis

**Figure 9:**
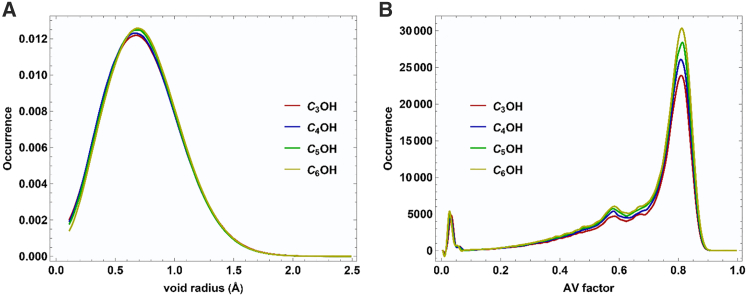
Void distribution analysis in MFF-alcohol system at x_1_ = 0.5 (A) Distribution of cavities relative to their radius. (B) Frequency distribution of the cavities’ isoperimetric ratio (AV factor).

To better understand the microscopic environment that governs molecular diffusion, we analyzed the distribution of free volume within the MFF-alcohol mixtures using two metrics: (i) the void (cavity) radius distribution and (ii) the isoperimetric ratio (AV factor), which characterizes the sphericity of voids.^18^^,^^19^ Figure 9A shows the probability distribution of void radii in equimolar mixtures (x_1_ = 0.5). All systems exhibit a similar profile, with a dominant peak centered around 0.7 Å, indicating a characteristic cavity size prevalent across the different alcohols. A slight increase in the peak height with increasing alkyl chain suggests a higher prevalence of voids in mixtures containing bulkier 2-alkanol. This trend can be attributed to the increasing steric hindrance and packing efficiency of longer-chain 2-alkanols.

**Figure 10:**
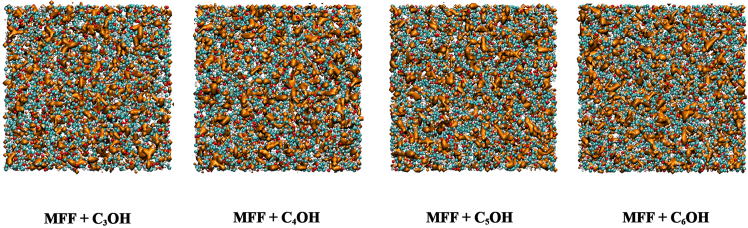
Snapshots visualize void spheres with 0.7 Å radius (orange region)

Figure 9B provides the distribution of the AV factor, a geometric descriptor of void compactness, where values closer to 1 indicate more spherical cavities. The dominant peak near AV ≈ 0.8 across all systems confirms that the majority of voids are nearly spherical in shape. The AV factor distribution exhibits a slight shift toward higher occurrence values in the mixtures with longer-chain alcohols (C_6_OH), indicating that the cavities in these systems possess greater geometric regularity. In order to visualize how distributed the local free volume, we generated void-sphere representations (radius 0.7 Å) for each mixture. These snapshots are shown in Figure 10. These void spheres highlight regions in the liquid where no atomic centers penetrate, serving as a qualitative map of free-volume pockets.

### Density and excess molar volume (Vᴱ)

**Figure 11:**
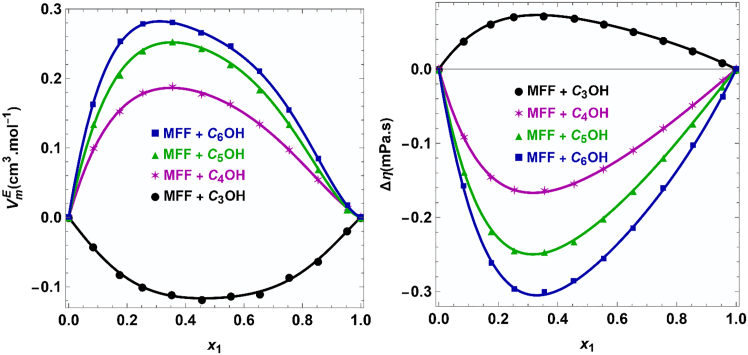
Excess molar volume and viscosity deviations for 5-methyl furfural (MFF) and 2-alkanol at 293.15 K

Density and viscosity of the binary systems of 5-methylfurfural and 2-alkanols, measured across the entire composition range, are presented in Tables S1, S2, S3 and S4. Using the obtained data, excess molar volumes were computed and listed in Tables S5, S6, S7 and S8. The behavior of Vᴱ across the composition range is illustrated in Figure 11. A notable behavior is observed: the binary system of 5-methylfurfural with 2-propanol exhibits negative VE values, while mixtures with all other 2-alkanols show positive VE values at the measured temperatures.

5-methylfurfural, which possesses a polar carbonyl group (C=O) within its aldehyde function, acts as a hydrogen bond acceptor. This group engages in intermolecular hydrogen bonding with the hydroxyl (-OH) group of the 2-alkanols. Furthermore, significant dipole-dipole interactions arise from the polar aldehyde group of 5-methylfurfural and the hydroxyl group of the 2-alkanols, complemented by London dispersion forces between the non-polar moieties of the molecules.

The negative VE values for the 2-propanol system signify that the mixture occupies a smaller volume than the sum of its pure components. This suggests either the formation of stronger intermolecular interactions or a more efficient interstitial accommodation of molecules in the mixture. In this specific case, the disruption of the hydrogen-bonded networks present in pure 2-propanol is more than compensated for by strong, highly directional hydrogen bonds and dipole-dipole interactions with 5-methylfurfural. The compact structure of 2-propanol results in minimal steric hindrance, allowing for an optimal geometric fit that promotes tight molecular packing and leads to volume contraction.

Conversely, the positive VE values observed for mixtures with 2-butanol and higher 2-alkanols indicate that these mixtures occupy a greater volume than anticipated. This reflects less efficient molecular packing and weaker net intermolecular forces relative to the pure components. For these systems, the primary effect upon mixing is the disruption of the robust, self-associated hydrogen-bonded structures of the pure 2-alkanols. The newly formed hydrogen bonds between 5-methylfurfural and the longer-chain 2-alkanols are less effective at minimizing volume. This disparity arises because the increasing size of the alkyl chain introduces significant steric barriers, preventing the close approach necessary for strong interactions and leading to a more disordered and expanded liquid structure.

With the increasing chain length of the 2-alkanol from 2-butanol onwards, the alkyl group becomes progressively bulkier, which enhances steric hindrance. This effect increasingly impedes efficient packing and weakens the overall interaction strength. Consequently, the disruption of the alcohol’s structure becomes more pronounced, causing the positive VE values to increase with the chain length of the 2-alkanol.

### Viscosity and deviations

Viscosity deviations (*Δη*), calculated from the experimental data in Tables S1, S2, S3 and S4, are shown in Figure 11. The results present a clear division: *Δη* is positive for the 5-methylfurfural and 2-propanol system but negative for all other 2-alkanol mixtures investigated. The positive *Δη* values for the 2-propanol mixture indicate that its viscosity is greater than the ideal mole fraction average, suggesting reduced molecular mobility and stronger intermolecular forces in the mixture. This observation is consistent with the negative VE values. The formation of strong, specific adducts between 5-methylfurfural and 2-propanol creates a more structured liquid arrangement that increases the resistance to flow. The efficient packing and strong attractive forces restrict the movement of the molecules, thus elevating the viscosity of the mixture.

In contrast, the negative *Δη* values for the higher 2-alkanol systems signify that the mixture’s viscosity falls below the ideal value. This implies a net reduction in flow resistance, which is attributed to weaker overall intermolecular interactions in the mixture compared to the pure constituents. In the pure state, 2-alkanols exhibit high viscosity due to their extensive hydrogen-bonded networks. Upon mixing with 5-methylfurfural, the disruption of these networks, combined with the sterically hindered and therefore weaker cross-interactions, results in an increase in free volume. This enhanced free volume facilitates greater molecular mobility, which in turn diminishes the mixture’s viscosity and yields negative *Δη* values, a behavior that aligns with the observed positive VE. As the 2-alkanol chain length extends from 2-butanol to 2-hexanol, the growing alkyl chain introduces greater steric hindrance. This further weakens the specific interactions and disrupts packing. Consequently, the mixture’s structure becomes increasingly disordered, and the free volume expands, making it easier for molecules to move past one another. This causes the viscosity to decrease more markedly relative to the ideal mixture, rendering *Δη* increasingly negative with the elongation of the alkyl chain.

### Conclusion

This study advances the development of sustainable biofuels by systematically analyzing the thermophysical and molecular interaction properties of 5-methylfurfural (MFF) blended with 2-alkanols (C_3_–C_6_) through an integrated experimental and molecular dynamics (MD) approach. The results demonstrate that short-chain alcohols, particularly 2-propanol, form strong hydrogen-bonding networks with the aldehyde group of MFF, resulting in compact molecular packing. In contrast, longer-chain alcohols such as 2-hexanol introduce steric hindrance, improving miscibility with conventional fuels and increasing energy density, thereby optimizing combustion stability and efficiency in compression ignition (CI) engines.

The strong agreement between experimental viscosity and density measurements and MD simulations validates the predictive reliability of this combined methodology, offering a robust framework for rational fuel blend design. By linking molecular-scale hydrogen-bonding and packing behavior to macroscopic thermophysical properties, this work provides actionable guidelines for tailoring blend composition to meet performance, efficiency, and emission-reduction targets. Overall, these insights contribute to the global transition toward renewable energy systems by enabling the formulation of next-generation biofuels that balance efficiency with environmental sustainability. Future research should focus on evaluating long-term blend stability and scalability in real-world engine environments, as well as exploring catalytic upgrading pathways that further enhance the viability of furan-based fuels in advanced energy applications.

### Limitations of the study

Although insights from this work are relevant to practical fuel formulation, real combustion environments involve complex factors, such as high-temperature reaction pathways, atomization behavior, and interactions with conventional fuels not captured in the present analysis. Future studies incorporating engine-scale testing, multicomponent blends, and reactive MD approaches would help bridge the gap between molecular-scale understanding and applied biofuel performance.

## Resource availability

### Lead contact

Requests for further information and resources should be directed to and will be fulfilled by the lead contact, Mohammad Almasi (m.almasi@malayeru.ac.ir).

### Materials availability

This study did not generate new unique reagents.

### Data and code availability

• All data supporting the findings of this study are available within the article and its supplemental information. Additional raw data are available from the lead contact upon reasonable request.
• This article does not report original code.
• Any additional information required to reanalyze the data reported in this article will be considered for sharing by the lead contact upon request.

## Acknowledgments

We gratefully acknowledge the Caroon Laboratory of Oil (in Ahvaz) for graciously providing the necessary facilities and support that enabled the completion of our experimental work.

## Author contributions

M. Almasi: conceptualization, data curation, and writing – original draft. M. Vatanparast: methodology, molecular dynamic simulation, and writing – review and editing.

## Declaration of interests

The authors declare no competing interests.

## Declaration of generative AI and AI-assisted technologies in the writing process

During the preparation of this article, the authors employed AI-powered tools for language enhancement. Following this assistance, the authors conducted a thorough review and revision of the content and assume complete responsibility for the publication’s final form.

## STAR★Methods

### Key resources table

**Table:** 

REAGENT or RESOURCE	SOURCE	IDENTIFIER
5-methyl furfural	Merck	CAS No. 620-02-0
2-Propanol	Sigma-Aldrich	CAS No. 67-63-0
2-Butanol	Sigma-Aldrich	CAS No. 78-92-2
2-Pentanol	Sigma-Aldrich	CAS No. 6032-29-7
2-Hexanol	Sigma-Aldrich	CAS No. 626-93-7

### Experimental model and study participant details

This work did not include any experimental model or study participants.

### Method details

#### Density and viscosity measurements

In the present investigation, we employed high-purity chemicals, including 5-methyl furfural (MFF) and 2-alkanols, supplied by Merck and Sigma-Aldrich. These compounds exhibited a purity exceeding 99%, thereby discarding the requirement for further purification procedures. Experimental data of density and viscosity at different temperatures are reported in Tables S1, S2, S3 and S4. These measurements were conducted using an Anton Paar SVM 3000 viscometer, an apparatus incorporating dual rotating cylinders operating at differential velocities to deliver accurate density and viscosity determinations. The instrument employs sophisticated thermal regulation mechanisms, essential for ensuring precision, given that even minor temperature deviations may markedly affect outcomes. Calibration of the viscometer was rigorously validated prior to each experimental sequence using distilled, degassed water. To maintain chemical stability, all reagents were degassed and stored in light-shielding containers. We prepared and characterized ten distinct mixture compositions, with mass measurements performed on a Mettler AE 163 balance, offering an uncertainty of ±0.14 mg. The uncertainties in density and relative viscosity measurements established at 0.0005 g/cm^3^ and 0.05, respectively.

#### Simulation details

Molecular dynamics (MD) simulations were carried out to investigate the structural and interactional behavior of four binary systems, each composed of 5-methyl furfural (MFF) and an alcohol: 2-propanol (C_3_OH), 2-butanol (C_4_OH), 2-pentanol (C_5_OH), or 2-hexanol (C_6_OH). Using the GROMACS 2025 simulation software, all simulations were carried out under the conditions of 1 atm pressure and 293.15 K temperature.^20^ The mole fraction of 5-methyl furfural (x_1_) was systematically varied between 0.1 and 0.9, alongside simulations of the pure components. Periodic boundary conditions were applied in three dimensions to a cubic simulation box containing 400 molecules for each system. Initial molecular arrangements were constructed using the PACKMOL V21 package.^21^ Force field parameters for 5-methylfurfural are listed in Table S9.

The OPLS-AA all-atom force field^22^^,^^23^^,^^24^^,^^25^ was employed due to its proven accuracy in modeling organic compounds, including alcohols.^23^^,^^26^ In particular, previous evaluations by Kashefolgheta et al. demonstrated the superior performance of OPLS-AA in predicting the free energies of solvation, achieving good agreement with experimental data for small organic molecules, with an a root-mean-square error (RMSE) of 2.9 kJ mol^−1^, affirming its suitability for the current study.^27^

The Particle Mesh Ewald (PME) method was employed to account for electrostatic interactions, and a cutoff of 1.2 nm was used to both Lennard-Jones and real-space Coulombic interactions. Bond constraints were enforced using the LINCS algorithm. The simulation workflow began with steepest descent energy minimization, followed by a two-step equilibration protocol: (i) 4 ns under the NVT ensemble with velocity-rescaling thermostat at 293.15 K, and (ii) 4 ns under the NPT ensemble using the Berendsen barostat to regulate pressure at 1 bar. Production runs of 10 ns were conducted with a 2 fs time step, keeping the system at 293.15 K and 1 bar using the velocity-rescale thermostat and the Parrinello–Rahman barostat. Periodic boundary conditions were applied in all directions throughout the simulations to reduce finite-size effects. Hydrogen bonds were defined by the conventional geometric criterion (O–O ≤ 0.35 nm and H–O–O angle ≤30°) and analyzed using GROMACS “gmx hbond” tool. Representative simulation snapshots at an MFF mole fraction of x1 = 0.5 are presented in Figure 1.

### Quantification and statistical analysis

No methods were used to determine whether the data met the assumptions of the statistical approach.
