# Supplementary material for: Thermophysical properties of 5-methylfurfural with alcohol additives: Toward efficient and sustainable biofuel blends
Source: iScience. 2025 Dec 31;29(2):114591. doi: 10.1016/j.isci.2025.114591 (PMC12874438; doi:10.1016/j.isci.2025.114591)
Supplement: Document S1. Tables S1–S9 [file mmc1.pdf]

iScience, Volume *29*

Supplemental information

Thermophysical properties of 5-methylfurfural with alcohol additives: Toward efficient and sustainable biofuel blends

Mohammad Almasi and Morteza Vatanparast

Table S1. Measured density and viscosity of 5-methyl furfural (1) + 2-Propanol (2) mixtures at various temperatures and pressure 0.1 MPa.

		$\rho(g.cm^{-3})$		
x_1	T(K) =293.15	T(K) =303.15	T(K) =313.15	T(K) =323.15
0	0.7854	0.7768	0.7680	0.7588
0.0836	0.8200	0.8112	0.8022	0.7927
0.1744	0.8557	0.8466	0.8374	0.8277
0.2522	0.8847	0.8754	0.8661	0.8562
0.3526	0.9202	0.9108	0.9014	0.8913
0.4561	0.9548	0.9453	0.9357	0.9255
0.5553	0.9861	0.9765	0.9669	0.9566
0.6546	1.0159	1.0062	0.9965	0.9862
0.7549	1.0443	1.0345	1.0250	1.0146
0.8536	1.0709	1.0611	1.0515	1.0411
0.9533	1.0963	1.0865	1.0770	1.0666
1	1.1078	1.0980	1.0885	1.0781
		η (mPa.s)		
0	2.42	1.80	1.36	1.05
0.0836	2.437	1.832	1.401	1.091
0.1744	2.438	1.845	1.425	1.119
0.2522	2.428	1.848	1.438	1.138
0.3526	2.404	1.846	1.449	1.159
0.4561	2.375	1.839	1.458	1.177
0.5553	2.343	1.829	1.463	1.193
0.6546	2.308	1.817	1.467	1.206
0.7549	2.271	1.802	1.468	1.219
0.8536	2.232	1.784	1.467	1.229
0.9533	2.192	1.764	1.462	1.234
1	2.171	1.754	1.458	1.237

x_1 is the mole fraction of 5-methyl furfural in binary mixtures. Standard uncertainties: $u(x)$ = 0.001, u(T) = 0.03 K, $u(\rho)$ = 0.0005 g.cm^{-3}. $u_r(\eta)$ = 0.05, $u_r(p)$ = 0.01.

Table S2. Measured density and viscosity of 5-methyl furfural (1) + 2-Butanol (2) mixtures at various temperatures and pressure 0.1 MPa.

		$\rho(g.cm^{-3})$		
x_1	T(K) =293.15	T(K) =303.15	T(K) =313.15	T(K) =323.15
0	0.8067	0.7984	0.7898	0.7806
0.0853	0.8334	0.8247	0.8159	0.8064
0.1747	0.8614	0.8524	0.8434	0.8337
0.2538	0.8860	0.8769	0.8677	0.8579
0.3555	0.9174	0.9082	0.8988	0.8888
0.4549	0.9478	0.9384	0.9289	0.9188
0.5531	0.9774	0.9679	0.9584	0.9481
0.6536	1.0074	0.9978	0.9882	0.9779
0.7547	1.0372	1.0275	1.0179	1.0076
0.8532	1.0659	1.0561	1.0466	1.0362
0.9554	1.0952	1.0854	1.0759	1.0655
1	1.1078	1.098	1.0885	1.0781
		η (mPa.s)		
0	3.67	2.54	1.80	1.33
0.0853	3.45	2.399	1.715	1.280
0.1747	3.262	2.286	1.651	1.248
0.2538	3.127	2.211	1.614	1.232
0.3555	2.973	2.128	1.578	1.222
0.4549	2.833	2.057	1.549	1.218
0.5531	2.706	1.995	1.528	1.218
0.6536	2.580	1.938	1.509	1.219
0.7547	2.459	1.883	1.493	1.224
0.8532	2.342	1.830	1.478	1.229
0.9554	2.222	1.776	1.464	1.235
1	2.171	1.754	1.458	1.237

x_1 is the mole fraction of 5-methyl furfural in binary mixtures. Standard uncertainties: $u(x)$ = 0.001, u(T) = 0.03 K, $u(\rho)$ = 0.0005 g.cm^{-3}. $u_r(\eta)$ = 0.05, $u_r(p)$ = 0.01.

Table S3. Measured density and viscosity of 5-methyl furfural (1) + 2-Pentanol (2) mixtures at various temperatures and pressure 0.1 MPa.

		$\rho(g.cm^{-3})$		
x_1	T(K) =293.15	T(K) =303.15	T(K) =313.15	T(K) =323.15
0	0.8093	0.8012	0.7927	0.7840
0.0854	0.8317	0.8232	0.8145	0.8055
0.1762	0.8564	0.8476	0.8387	0.8295
0.2534	0.8779	0.8690	0.8599	0.8505
0.3553	0.9070	0.8979	0.8887	0.8791
0.4547	0.9361	0.9268	0.9175	0.9077
0.5529	0.9655	0.9561	0.9467	0.9367
0.6538	0.9964	0.9868	0.9774	0.9673
0.7549	1.0281	1.0184	1.0089	0.9987
0.8541	1.0600	1.0503	1.0407	1.0304
0.9528	1.0923	1.0825	1.0730	1.0626
1	1.1078	1.0980	1.0885	1.0781
		η (mPa.s)		
0	3.97	2.81	1.99	1.39
0.0854	3.678	2.610	1.861	1.314
0.1762	3.435	2.448	1.763	1.265
0.2534	3.270	2.348	1.707	1.241
0.3553	3.085	2.236	1.651	1.224
0.4547	2.920	2.143	1.605	1.215
0.5529	2.774	2.062	1.572	1.214
0.6538	2.630	1.989	1.541	1.215
0.7549	2.493	1.917	1.515	1.221
0.8541	2.360	1.849	1.492	1.226
0.9528	2.233	1.785	1.469	1.235
1	2.171	1.754	1.458	1.237

x_1 is the mole fraction of 5-methyl furfural in binary mixtures. Standard uncertainties: $u(x)$ = 0.001, u(T) = 0.03 K, $u(\rho)$ = 0.0005 g.cm^{-3}. $u_r(\eta)$ = 0.05, $u_r(p)$ = 0.01.

Table S4. Measured density and viscosity of 5-methyl furfural (1) + 2-Hexanol (2) mixtures at various temperatures and pressure 0.1 MPa.

		$\rho(g.cm^{-3})$		
x_1	T(K) =293.15	T(K) =303.15	T(K) =313.15	T(K) =323.15
0	0.8142	0.8061	0.7984	0.7901
0.0832	0.8328	0.8244	0.8164	0.8079
0.1768	0.8551	0.8464	0.8382	0.8294
0.2539	0.8745	0.8656	0.8572	0.8482
0.3551	0.9012	0.8921	0.8835	0.8743
0.4532	0.9284	0.9191	0.9104	0.9010
0.5547	0.9579	0.9485	0.9396	0.9300
0.6533	0.9881	0.9786	0.9695	0.9597
0.7541	1.0207	1.0111	1.0019	0.9919
0.8548	1.0551	1.0454	1.0361	1.0259
0.9552	1.0912	1.0814	1.0720	1.0617
1	1.1078	1.0980	1.0885	1.0781
		η (mPa.s)		
0	5.15	3.29	2.28	1.64
0.0832	4.744	3.042	2.116	1.536
0.1768	4.361	2.831	1.991	1.465
0.2539	4.097	2.688	1.914	1.421
0.3551	3.791	2.527	1.830	1.378
0.4532	3.514	2.382	1.754	1.342
0.5547	3.242	2.245	1.681	1.311
0.6533	2.989	2.122	1.616	1.284
0.7541	2.743	2.007	1.561	1.262
0.8548	2.501	1.897	1.509	1.245
0.9552	2.266	1.795	1.471	1.238
1	2.171	1.754	1.458	1.237

x_1 is the mole fraction of 5-methyl furfural in binary mixtures. Standard uncertainties: $u(x)$ = 0.001, u(T) = 0.03 K, $u(\rho)$ = 0.0005 g.cm^{-3}. $u_r(\eta)$ = 0.05, $u_r(p)$ = 0.01.

Table S5. Excess molar volumes and viscosity deviations of 5-methyl furfural (1) + 2-Propanol (2) mixtures at different temperatures.

$V_m^E (cm^3.mol^{-1})$				
x_1	T(K) =293.15	T(K) =303.15	T(K) =313.15	T(K) =323.15
0.0836	-0.042	-0.042	-0.039	-0.029
0.1744	-0.082	-0.072	-0.063	-0.054
0.2522	-0.100	-0.084	-0.076	-0.061
0.3526	-0.111	-0.100	-0.093	-0.074
0.4561	-0.118	-0.111	-0.093	-0.077
0.5553	-0.113	-0.106	-0.093	-0.078
0.6546	-0.110	-0.102	-0.083	-0.077
0.7549	-0.086	-0.076	-0.077	-0.068
0.8536	-0.063	-0.057	-0.050	-0.045
0.9533	-0.019	-0.018	-0.019	-0.017
$\Delta\eta$ (mPa.s)				
0.0836	0.038	0.036	0.033	0.025
0.1744	0.061	0.053	0.048	0.036
0.2522	0.071	0.060	0.053	0.041
0.3526	0.072	0.062	0.054	0.043
0.4561	0.069	0.060	0.053	0.042
0.5553	0.061	0.055	0.049	0.039
0.6546	0.051	0.047	0.043	0.034
0.7549	0.039	0.037	0.034	0.028
0.8536	0.025	0.023	0.023	0.019
0.9533	0.009	0.008	0.009	0.006

x_1 is the mole fraction of 5-methyl furfural in binary mixtures. Standard uncertainties *u* are *u* (T) = 0.03 K, $u(\rho)$ = 0.0005 g.cm^{-3}, $u(V_m^E)$ = 0.008 cm^3.mol^{-1}, $u(\Delta\eta)$ = 0.007 mPa.s, u_r(P) = 0.01, $u_r(\eta)$ = 0.05.

Table S6. Excess molar volumes and viscosity deviations of 5-methyl furfural (1) + 2-Butanol (2) mixtures at different temperatures.

x_1	T(K) =293.15	T(K) =303.15	T(K) =313.15	T(K) =323.15
		$V_m^E (cm^3.mol^{-1})$		
0.0853	0.099	0.127	0.137	0.157
0.1747	0.152	0.194	0.214	0.240
0.2538	0.179	0.216	0.246	0.269
0.3555	0.188	0.217	0.255	0.283
0.4549	0.177	0.209	0.246	0.270
0.5531	0.163	0.190	0.217	0.249
0.6536	0.134	0.156	0.184	0.204
0.7547	0.097	0.115	0.137	0.146
0.8532	0.054	0.069	0.075	0.085
0.9554	0.013	0.017	0.019	0.022
		$\Delta\eta$ (mPa.s)		
0.0853	-0.092	-0.074	-0.056	-0.042
0.1747	-0.146	-0.117	-0.089	-0.066
0.2538	-0.163	-0.130	-0.099	-0.074
0.3555	-0.164	-0.133	-0.100	-0.075
0.4549	-0.155	-0.125	-0.095	-0.070
0.5531	-0.135	-0.110	-0.083	-0.061
0.6536	-0.110	-0.088	-0.067	-0.050
0.7547	-0.08	-0.064	-0.049	-0.036
0.8532	-0.049	-0.039	-0.030	-0.022
0.9554	-0.016	-0.013	-0.009	-0.006

x_1 is the mole fraction of 5-methyl furfural in binary mixtures. Standard uncertainties *u* are *u* (T) = 0.03 K, $u(\rho)$ = 0.0005 g.cm^{-3}, $u(V_m^E)$ = 0.008 cm^3.mol^{-1}, $u(\Delta\eta)$ = 0.007 mPa.s, u_r(P) = 0.01, $u_r(\eta)$ = 0.05.

Table S7. Excess molar volumes and viscosity deviations of 5-methyl furfural (1) + 2-Pentanol (2) mixtures at different temperatures.

		$V_m^E(cm^3.mol^{-1})$		
x_1	T(K) =293.15	T(K) =303.15	T(K) =313.15	T(K) =323.15
0.0854	0.135	0.170	0.184	0.207
0.1762	0.206	0.258	0.284	0.313
0.2534	0.241	0.288	0.326	0.362
0.3553	0.254	0.301	0.336	0.373
0.4547	0.244	0.293	0.325	0.363
0.5529	0.221	0.260	0.290	0.330
0.6538	0.185	0.224	0.243	0.273
0.7549	0.135	0.166	0.184	0.205
0.8541	0.070	0.083	0.102	0.115
0.9528	0.012	0.019	0.021	0.028
		$\Delta\eta$ (mPa.s)		
0.0854	-0.138	-0.110	-0.084	-0.063
0.1762	-0.218	-0.176	-0.133	-0.098
0.2534	-0.244	-0.194	-0.148	-0.110
0.3553	-0.246	-0.199	-0.150	-0.112
0.4547	-0.232	-0.187	-0.143	-0.105
0.5529	-0.201	-0.164	-0.124	-0.091
0.6538	-0.164	-0.131	-0.101	-0.075
0.7549	-0.119	-0.096	-0.073	-0.054
0.8541	-0.073	-0.059	-0.044	-0.033
0.9528	-0.023	-0.019	-0.014	-0.009

x_1 is the mole fraction of 5-methyl furfural in binary mixtures. Standard uncertainties u are u (T) = 0.03 K, $u(\rho)$ = 0.0005 g.cm^{-3}, $u(V_m^E)$ = 0.008 cm^3.mol^{-1}, $u(\Delta\eta)$ = 0.007 mPa.s, u_r(P) = 0.01, $u_r(\eta)$ = 0.05.

Table S8. Excess molar volumes and viscosity deviations of 5-methyl furfural (1) + 2-Hexanol (2) mixtures at different temperatures.

		$V_m^E (cm^3.mol^{-1})$		
x_1	T(K) =293.15	T(K) =303.15	T(K) =313.15	T(K) =323.15
0.0832	0.162	0.190	0.219	0.230
0.1768	0.253	0.303	0.339	0.371
0.2539	0.278	0.337	0.382	0.421
0.3551	0.280	0.340	0.387	0.424
0.4532	0.265	0.327	0.361	0.396
0.5547	0.246	0.295	0.330	0.361
0.6533	0.210	0.248	0.283	0.310
0.7541	0.154	0.181	0.203	0.225
0.8548	0.084	0.101	0.111	0.127
0.9552	0.017	0.025	0.024	0.025
		$\Delta\eta$ (mPa.s)		
0.0832	-0.158	-0.120	-0.096	-0.070
0.1768	-0.262	-0.187	-0.144	-0.104
0.2539	-0.297	-0.212	-0.157	-0.117
0.3551	-0.301	-0.218	-0.158	-0.119
0.4532	-0.286	-0.212	-0.153	-0.115
0.5547	-0.256	-0.193	-0.143	-0.105
0.6533	-0.215	-0.165	-0.127	-0.093
0.7541	-0.161	-0.125	-0.099	-0.074
0.8548	-0.103	-0.08	-0.068	-0.051
0.9552	-0.038	-0.028	-0.024	-0.017

x_1 is the mole fraction of 5-methyl furfural in binary mixtures. Standard uncertainties *u* are *u* (T) = 0.03 K, $u(\rho)$ = 0.0005 g.cm^{-3}, $u(V_m^E)$ = 0.008 cm^3.mol^{-1}, $u(\Delta\eta)$ = 0.007 mPa.s, u_r(P) = 0.01, $u_r(\eta)$ = 0.05.

Table S9. Force Field Parameters for 5-Methylfurfural

Atom No.	Element	Partial Charge (e)	σ (nm)	ε (kJ/mol)	Mass (g/mol)
1	H	+0.0311	0.250	0.1255	1.008
2	C	+0.4701	0.355	0.2929	12.011
3	C	–0.0483	0.355	0.2929	12.011
4	O	–0.5033	0.296	0.8786	15.999
5	O	–0.2261	0.290	0.5858	15.999
6	C	–0.0801	0.355	0.2929	12.011
7	C	+0.4193	0.355	0.2929	12.011
8	H	+0.1773	0.242	0.1255	1.008
9	C	–0.3939	0.355	0.2929	12.011
10	C	–0.5214	0.350	0.2761	12.011
11	H	+0.1993	0.242	0.1255	1.008
12	H	+0.1587	0.250	0.1255	1.008
13	H	+0.1587	0.250	0.1255	1.008
14	H	+0.1587	0.250	0.1255	1.008
